# A health impact assessment of the 2014 Commonwealth Games in Glasgow

**DOI:** 10.1016/j.puhe.2010.04.004

**Published:** 2010-08

**Authors:** G. McCartney, S. Palmer, J. Winterbottom, R. Jones, R. Kendall, D. Booker

**Affiliations:** a MRC Social and Public Health Sciences Unit, 4 Lilybank Gardens, Glasgow G12 8RZ, UK; b Glasgow City Council, Glasgow, UK; c Glasgow Centre for Population Health, Glasgow, UK; d NHS Greater Glasgow and Clyde, Glasgow, UK

**Keywords:** Health impact assessment, Glasgow, Commonwealth Games, Sport, Economy, Culture

## Abstract

**Objective:**

To influence the planning of the 2014 Commonwealth Games such that the positive impacts are maximized and the negative impacts are mitigated.

**Study design:**

Participatory health impact assessment (HIA).

**Methods:**

A participatory HIA was performed using standard World Health Organization methods. A scoping event was held to involve decision makers in the process and to identify the key areas for consideration. A large community engagement exercise and a systematic review were conducted as part of the evidence-gathering phase. The results of the HIA were reported to the key decision makers involved in the Glasgow City Council legacy strategy.

**Results:**

The likely net health impact of hosting the Commonwealth Games was uncertain. It was suggested that the main mechanisms through which impacts were likely to be felt were: the economy; civic pride; engagement in decision making; the provision of new infrastructure; and participation in cultural events. A series of recommendations was produced in order to maximize positive health benefits and mitigate negative impacts.

**Conclusions:**

HIA is a useful tool for engaging communities and decision makers in the public health agenda. HIAs of major multi-sport events are limited by a lack of quality evidence and the inability to predict impacts reliably.

## Introduction

On 9 November 2007, it was announced that the city of Glasgow was to host the 2014 Commonwealth Games. The bid put together by Glasgow City Council and the Scottish Government highlighted a range of benefits that playing host would bring to the local population.^1^ This included a range of ‘legacy’ benefits encompassing the familiar determinants of health (employment, housing etc.) as well as explicit health and wellbeing outcomes:

*“This investment will…contribute to the key objectives of improving the health of our population particularly around physical activity and the prevention of obesity. These in turn will contribute also to overall levels of confidence, wellbeing and mental health…”*^1^

Following the announcement that Glasgow had won its bid, a process of designing legacy plans commenced for the City Council and Scottish Government. These were to be the detailed mechanisms through which the benefits outlined in the bid document were to be realized.^2^ This was similar to the process adopted for the 2012 Olympics in London.^3^ A group of public health professionals and policy makers advocated for a health impact assessment (HIA) to be undertaken as an explicit attempt to influence those plans, such that the potential health benefits of playing host might be maximized and any negative impacts mitigated. This was proposed to fit into the time scale for the drafting of the legacy documents (with a particular focus on the Glasgow City Council legacy plan).^4^

The hosting of major sports events can be controversial, particularly where there are perceived to be harmful or unwanted outcomes.^5–9^ In Glasgow, the most deprived and unhealthy city in the UK,^10,11^ there is a particular need for policy and interventions to improve health. The Commonwealth Games is seen by policy makers to be part of this effort, and it is for public health professionals to advise on how a positive health legacy can be best realized. Hosting major events is not a remedy for all of Glasgow’s health and social ills, particularly since the city has a long history of such activity (including the 1988 Garden Festival, 1990 City of Culture and 1999 City of Architecture and Design)^5,12^ without a step change in its fortunes.^10^ However, Glasgow City Council and the Scottish Government consider that the Games have the potential to have a significant impact, and significant resources have been committed to hosting the event.^1^

HIA is an important tool to encourage evidence-informed policy making in favour of health.^13^ It is limited by the quality and breadth of the evidence base upon which to make recommendations and a lack of studies evaluating the effectiveness of HIA in predicting outcomes.^14,15^ This HIA is the start of a process to predict the impact of hosting the Games on health and the determinants of health; to influence the planning of the Games and the associated legacy programmes; and to evaluate the actual impact of the event (thereby facilitating a comparison between predicted and actual outcomes).

## Methods

### Screening

The standard World Health Organization framework for conducting HIAs was followed.^16^ The first opportunity to perform an HIA arose following the decision to award the Games to Glasgow, and so the purpose of the HIA was not to guide decision makers on whether or not a bid should be entered, but instead to influence the resulting legacy plans based on the information given in the bid document.^1^ A multi-agency group was formed to discuss the possibility as a proxy for the HIA screening stage, and a recommendation to undertake a participatory HIA was approved by the City Council as a means to inform its Games legacy plan. The geographical boundary of the HIA was agreed to be the City of Glasgow.

### Scoping

A scoping event was held in August 2008 in Celtic Park (the venue for the Games opening ceremony) involving 120 stakeholders. These included elected councillors, council officials and representatives of various other organizations (e.g. housing associations, NHS Greater Glasgow and Clyde, Culture and Sport Glasgow, the Scottish Government, academics and community representatives). The participants at this event were encouraged during a series of interactive workshops to consider all the potential health impacts of the Games, the size of the impact, the groups most likely to be affected, and the potential for influencing decisions pertaining to the impact. This was in order to prioritize potential impacts for further assessment. The scoping event, and the discussions pertaining to it, also facilitated a process of engaging with decision makers such that the findings of the assessment could be produced in a timely and relevant manner.^17^

### Evidence gathering

The key areas of impact identified at the scoping event were used to develop questions for public consultation. Evidence was gathered from the community utilizing an extensive community engagement exercise (described in Box 1), and from other events using a systematic review^18^ and discussion with the evaluators from the 2002 Commonwealth Games in Manchester. This evidence was reflected back to the community as a further element of the community engagement exercise; a process which formed the stakeholder involvement phase of the HIA. This also provided an opportunity for community involvement in the appraisal of the evidence and in the formulation of the recommendations.

### Evidence appraisal

Recommendations were formulated by the HIA steering group using the evidence gathered as part of the HIA following the appraisal and community engagement. Where possible, account was taken of existing and planned activities in the city.

### Reporting

The full report of the HIA process and recommendations was presented to all the relevant stakeholders (including elected councillors, city council officials and the 2014 Games organizing committee) in time for this to be reflected in the legacy plans and in advance of publication. A summarized version of the HIA report was distributed to the public shortly after the publication of the City’s legacy brochure.^19^

Only one part of the evaluation phase of the HIA has been completed; an evaluation of the community engagement strategy for the HIA. The evaluations of the impact of the HIA on legacy planning, and of the HIA process, are yet to be completed. The evaluation plans for the overall impact of the Games on health and the determinants of health are emerging but are beyond the scope of the HIA. All the supporting documentation relating to the HIA method is available on the Internet (http://www.gcph.co.uk/content/view/167/143/).

## Results

### Scoping

The key areas of potential impact identified from the scoping event were: employment and employability; the impact on Glasgow’s image; regeneration; civic pride; health and wellbeing; infrastructure development; the environment; and a number of cross-cutting themes such as community engagement, tackling inequalities and community cohesion. It was unclear whether the impact on each of these areas was likely to be positive or negative (i.e. the Games were described by participants as both a threat and an opportunity to tackle inequalities). Engagement with senior decision makers within the city was achieved, and the event prepared them to receive the HIA recommendations which they would be expected to implement in due course.

### Evidence gathering

#### Glasgow Household Survey^22^

The survey showed that residents believed the Games would have a positive impact on them, their families, their local area and Glasgow as a whole. Those living closest to the planned Games village were less likely to believe that there will be a positive impact on themselves and their families. For Glasgow to benefit as much as possible from the Games, the priorities, according to residents, were to improve the image of Glasgow and to provide access to employment opportunities associated with the Games.

#### ‘Have your say’ workshops

The key areas of potential impacts identified from the workshops included employment and employability, public transport, crime and security, and improved facilities for physical activity. A desire for enhanced community engagement, a reduction in inequalities, social inclusion and community cohesion were also expressed.

#### ‘Have your say’ questionnaire^21^

There were a total of 1640 electronic responses and 274 paper returns of the questionnaire. The analysis of these responses indicated that boosting civic pride and the cultural programmes attached to the Games were particularly important to respondents. Many thought that promoting a ‘feel good’ factor would be the strongest legacy of the Games. It was perceived that a key legacy would be improved sports facilities in terms of their accessibility and suitability. However, people felt that in order for the Games to have a lasting legacy, the local community would need to be actively engaged throughout the planning and delivery of the Games. Seventy-five percent of those answering the questionnaire expressed a desire to be involved in some capacity.

#### Systematic review^18^

A systematic review of the impacts of major multi-sport events (1978–2008) on the health and determinants of health was performed. The interim findings were reported to the HIA steering group and were included in the community feedback events. Fifty-four studies were included in the review, but the quality of the evidence was low and there were gaps in the outcomes examined. Five studies reported health outcomes from previous events. These reported that: paediatric and illicit-drug-induced hospital presentations increased; childhood asthma hospital admissions decreased; and suicide rates were unchanged. Economic impacts were unclear because of the use of estimated data beyond the date of the event, but there were studies showing positive and negative impacts on economic growth and employment. The review concluded that the organizers of future events would need to focus on generating health and socio-economic benefits together with a robust evaluation framework if they were to demonstrate any impacts after the event.

### Evidence appraisal

The evidence available suggested that the Games were likely to impact on a wide range of the determinants of health. This included relatively ‘hard’ outcomes such as the economy, and ‘soft’ outcomes such as civic pride. The net impact on health was impossible to estimate, but potential impacts on particular determinants of health were identified (Table 1), although it was difficult to predict the likelihood of these impacts (either positive or negative) being realized.

#### Community engagement feedback events

These events provided feedback to local communities and stakeholders on the findings of the evidence gathering and appraisal, and verified that the impacts identified were appropriate.

### Recommendations and reporting

The summarized recommendations arising from the HIA are shown in Table 1 (full details of the recommendations and the evidence underpinning them are available in the full report).^23^ The potential impacts of the plans are uncertain and can be seen (and framed) as opportunities or threats. For example, the planning of new sports facilities can be seen as an opportunity for the community to be empowered through being involved in their design, or can be seen as a threat to community empowerment if infrastructure is perceived to be imposed on a community without their involvement or consent. This tension is present in all of the potential impacts of the plans (Table 1). However, a series of clear recommendations was more readily developed for improving the potential impacts of hosting the Games. This drew upon existing strategic plans and the policy context in Glasgow. Thus, even where the overall impact on employment, for example, was uncertain, it was possible to suggest policy modifications that would maximize the positive impact on health and health inequalities.

## Discussion

### Main finding of this study

The impact of hosting the 2014 Commonwealth Games on the health of Glaswegians, and the determinants of their health, is uncertain. There are high public and governmental expectations of playing host, and the Games have generated a great deal of interest and debate about the possible impacts. A range of recommendations have been outlined (the recommendations contained within the full report are more specific, achievable and measurable than those in the summarized version outlined in Table 1) that reflect the available evidence and collective wisdom of the public and participants in the HIA process. Careful evaluation is required to determine whether these impacts are realized and whether the HIA process has influenced the decision-making process. It is likely that the Games will mainly influence health through impacts on the economy, civic pride, engagement in decision making, the provision of new infrastructure, and participation in cultural events. It was challenging to provide accurate estimates of the effects because of a lack of quality evidence from similar interventions.

The HIA community engagement process was evaluated using the National Standards for Community Engagement and the VOiCE (Visioning Outcomes in Community Engagement) tool with support from the Scottish Community Development Centre. The final score was 5 (out of a possible 6) indicating a ‘very good’ performance with major strengths in relation to the National Standards, and also in terms of achieving the stated outcomes of the work. The main strengths were in relation to planning, using a range of methods, working together, sharing information and providing feedback. The main deficits were that some elements of the engagement were rushed, and it was not certain that a representative cross-section of the community was reached. The evaluation found that the community engagement was successful in raising awareness of the delivery plans for the Games, the potential health impacts of the Games, and the National Standards for Community Engagement. Individuals involved in the process developed an increased sense of ‘connectedness’ to the Games, and some also gained skills and experience in community engagement. This has left people with increased capacity to participate in any further community engagement opportunities. The community engagement work undertaken as part of the HIA has had a clear influence on the community engagement and consultation strategy being developed by the Council’s legacy team.

### What is already known on this topic

HIA is an established mechanism for public health professionals to inform policy making with the available evidence and expertise. This is particularly important for social interventions not aimed specifically at generating health effects, which are likely to be an important influence on health but may not have sufficient health input into their planning and conduct. There are precedents for conducting HIAs on major multi-sports events,^45^ and there may be an increasing desire for quality public health input to policy making.^46^

### What this study adds

The impact of major multi-sport events on health and the determinants of health is unknown, and the 2014 Games cannot be expected to solve all of Glasgow’s health challenges.^10^ However, HIA can be used to engage with the public and policy makers such that the health agenda is made more explicit and high profile. It may be that the Games can act as a catalyst to support existing aims around health, and can help to focus efforts of a wide range of organizations on such challenges. It is clear that the Glasgow public are keen to be involved in planning the Games legacy and that, when given opportunities to be involved, they provide a useful and unique insight.

### Limitations of this study

As with all HIAs, the value of this work is limited by the evidence base upon which it draws, the inability to predict impacts accurately and with certainty, and the extent to which its recommendations are acted upon.^14^

The survey methods used to gather the opinions of residents have the potential for selection bias. For example, the Glasgow Household Survey sampling method involves selecting addresses in an area, and if there is no response, sampling from nearby dwellings. Similarly, the ‘Have your say’ questionnaire was open to selection bias because of its ‘opt-in’ nature (responses were gathered from Internet users on the Glasgow City Council website and from postal responses to questionnaires distributed in public buildings across the area). This potential for bias was less important in the generation of a list of possible impacts than it was for determining public priorities.

## Figures and Tables

**Table 1:** Summarized recommendations of the health impact assessment.

	Evidence appraisal	Potential impacts on health or the determinants of health	Recommendations
Infrastructure (facilities)	• The long-term viability of facilities and accessibility was prioritized by the public (especially relating to cost, physical access and transport to facilities)^17,21,24–26^ • The need for increased capacity for public use was highlighted following the 2002 Games^27^ • Access to affordable, healthy food within the new sporting facilities was highlighted by the public^21^	• Increased physical activity • Limited accessibility (in terms of physical access, transport and cost)	• New facilities should be accessible to local people and meet their needs in years to come
Infrastructure (transport)	• There was some public support for the creation of a sustainable and comprehensive transport system^2,7,17,21^ • There was concern that new roads would divide communities, lead to accidents and create pollution^7,20–26^ • The plans should enhance active travel^2,7,17,21,24^ • There was concern about possible congestion during the event^17,21^	• Increased noise pollution, air pollution, community severance, traffic accidents and congestion • Modal shift towards active transport	• Disruption during construction and the Games should be minimized • Accessible and user-friendly transport should be developed as part of the plans
Civic pride and city image	• Civic pride is perceived to be the main benefit of playing host^21,28^ • It was a public priority to use this opportunity to improve Glasgow’s image^17,24,29,30^	• Increased civic pride • Increased tourism and trade • Negative publicity for the city and its people	• The community should be involved in the promotion of Glasgow as a friendly city • A strategy to improve the city’s image should be developed
Health and well-being (individual behaviour change)	• The public perceived an opportunity for increased physical activity,^21,29,31,32^ increased access to healthy food,^17,21,29^ and reduced alcohol and tobacco consumption,^31,33^ but there were concerns that these opportunities would be unequally spread^17,18^ • There was concern that Games sponsorship could undermine health promotion messages^17^	• Increased health inequalities • Increased physical activity, increased access to healthy food and reduced smoking • Increased alcohol use	• Use opportunities to increase healthy eating, smoke-free environments and physical activity (including safer active travel)
Housing and public space	• The Games village was expected to be an important legacy with potential for positive and negative impacts for the existing and incoming residents^7,17,24,26,29^	• Creation of a sustainable, cohesive and vibrant new community • Gentrification and social division with existing community in Dalmarnock • Rising housing costs	• Use healthy and sustainable urban design principles • Involve the local community in decision making around the Games village • Create an appropriate mix of social and private housing in the Games village
Participation in cultural and sporting events	• The public were keen to develop a cultural legacy for all parts of the community^24,29,30,33^ • A well-designed cultural programme was believed to be able to empower and educate^21,29^	• Increased pride, empowerment and cultural awareness • Reduced crime	• Involve local people in event planning • A brand logo should be provided for community use
Economy and employment	• The creation of sustainable jobs and skills for local people was a public priority^17,21,26,29^ • Procurement was identified as an opportunity to stimulate the local economy and promote ethical and sustainable business^17,21,26,29^ • The cost of the event was a concern including the potential for funds to be diverted from other services^17,21,34^	• Increased employment and tourism • Employment opportunities unequally distributed and short term	• Locals should be given support to access employment and training opportunities • Small businesses should be supported in bidding for Games contracts • The Games budget should be transparent and the impact on services minimized
Volunteering	• Volunteering was identified as a route to increasing employability^21^ • The experience of volunteering at other events was mixed^35–39^ • There was evidence that volunteers could be encouraged by being part of a ‘big event’, personal development goals, and the promise of meeting new people^21^	• Increased employability • Increased volunteering in the city after the event • Inequality in the uptake of volunteering opportunities	• Local people should be supported to access volunteering opportunities • Volunteers should receive expenses and training (linked to employability)
Community safety, antisocial behaviour and crime	• The Games are perceived as an exacerbating factor for crime and antisocial behaviour, but also an opportunity for improvement (particularly with respect to diversionary activities)^24,30–33,40,41^ • There is potential for an increase in substance misuse, particularly around the closing ceremony^17,42^ • The Games were seen as an opportunity to increase the cleanliness of the streets and enhance toilet facilities • Evidence from a previous event suggests that demand for police services will increase^43^	• Increased alcohol-related antisocial behaviour • Increased crime • Cleaner streets	• Alcohol licensing laws should be strictly enforced • The opportunity for improved cultural awareness should be utilized • A detailed crime reduction policy for the Games should be planned
Community engagement	• There was clear public demand for community involvement in Games-related decision making^21^ • The National Standards on Community Engagement were identified as a useful tool to ensure adequate public involvement^44^	• Communities are engaged and empowered	• The National Standards of Community Engagement should be implemented and independently evaluated for all aspects of the Games planning
Sports development legacy	• Developing a sports legacy was not a public priority, although a grassroots sports legacy was seen to be more important than that for elite athletes^17,21,24^ • There was a minority view that sport could be used to engage excluded groups^17,21,24^	• Increased sports participation • Increased inequalities in sports participation	• Grassroots sports participation should be prioritized through increased coaching and facilities for the general public
Environment, sustainable development and carbon footprint	• The Games were identified as an opportunity to develop sustainable procurement, waste management, reduce air pollution and improve the urban environment^7,17,21,24,29,31^ • The construction of facilities was recognized as a potential source of noise and air pollution^17^	• Environmental improvements (urban drainage, transport design, new village housing) • Improved procurement • Noise and air pollution	• Sustainability should be embedded into all Games-related projects • The Games should be used to showcase environmentally-friendly practice especially related to the design of the village
Monitoring and evaluation	• The evidence base for the impacts of major sports events is of poor quality and is sparsely populated^18^	• Future events are able to learn from Glasgow’s experience	• Robust evaluations of the HIA process, influence of the HIA and impact of the Games should be undertaken

## Box 1 Health impact assessment (HIA) community engagement strategy

The community engagement strategy targeted community members in preference to the decision makers targeted in the scoping stage. It aimed to provide an opportunity for people to question the potential impacts identified during the scoping event, suggest additional or alternative impacts, and suggest interventions to mitigate negative impacts and maximize positive impacts. The National Standards for Community Engagement were adopted as the quality standard for this process.^20^ The findings from previous community engagement exercises in the city were collated and fed into the evidence appraisal stage.

**Presentations to existing community groups**
The existing community engagement structures within the city were used to distribute information about the HIA and mechanisms for becoming involved (facilitated by the community planning partnerships and community engagement coordinating groups). Presentations were made to all 13 community reference groups (representative panels of local residents) across the city to raise awareness of the work.

**Glasgow Household Survey**
Specific Games-related questions were designed and added to the Glasgow Household Survey (a routine, twice-yearly, independent survey of 1000 city residents).

**‘Have your say’ workshops**
A series of ‘Have your say’ workshops were organized in communities across the city, using existing and newly trained participatory appraisal facilitators. Eighteen events (with a total of 42 discussion groups) were held in total and attended by over 350 community members. This included workshops with targeted youth groups and equality groups.

**‘Have your say’ questionnaire^21^**
The ‘Have your say’ questionnaire was specifically developed for the HIA and based on the themes which emerged from the HIA scoping phase. The electronic version of a ‘Have your say’ questionnaire was posted on the Glasgow City Council website between October 2008 and the end of December 2008. Seventeen thousand paper copies of the questionnaire were distributed via general practitioner and dentist surgeries, local housing organizations, Culture and Sport Glasgow community venues, libraries, sport and leisure centres and museums, and City Council premises.

**Community engagement feedback events**
Following these initial community engagement activities, five events were held in each of Glasgow’s strategic planning areas in March 2009 to provide feedback to local communities and stakeholders on the evidence appraisal, and to verify that the issues identified were appropriate.
